# A New Topology-Switching Strategy for Fault Diagnosis of Multi-Agent Systems Based on Belief Rule Base

**DOI:** 10.3390/e24111591

**Published:** 2022-11-02

**Authors:** Ziyi Wang, Shaohua Li, Wei He, Ruohan Yang, Zhichao Feng, Guowen Sun

**Affiliations:** 1 School of Computer Science and Information Engineering, Harbin Normal University, Harbin 150500, China; 2 School of Innovation and Entrepreneurship, Dalian University of Foreign Languages, Dalian 116044, China; 3 High-Tech Institute of Xi’an, Xi’an 710025, China; 4 School of Electronics and Information, Northwestern Polytechnical University, Xi’an 710072, China

**Keywords:** belief rule base (BRB), multi-agent system (MAS), fault diagnosis, topology switching

## Abstract

Effective fault-diagnosis strategies have been the focus of research on multi-agent systems (MASs). In this paper, the belief rule base (BRB)-based distributed fault-diagnosis problem for MASs is investigated, and a topology-switching strategy is developed to increase the reliability of fault-diagnosis model. Firstly, a BRB-based distributed fault-diagnosis model is constructed for the MAS with multiple faults, then expert knowledge is used to judge whether the agent is faulty. Then, considering that the system may be influenced by the fault or some other factors and thus leading to a decrease in the accuracy of the fault-diagnosis results, a topology-switching strategy based on the average distance of the output diagnosis accuracy is proposed to update the topology of the agent so that the fault-diagnosis results can be more reliable. Note that the topology-switching threshold is designed based on the average distance between the accuracy of the fault diagnosis of each agent. The method proposed in this paper can solve the problem when the fault-diagnosis accuracy of the model is affected by some common factors and thus decreases, and can improve the reliability of the fault-diagnosis model very well. Finally, the effectiveness of the BRB-based distributed fault-diagnosis model and the proposed topology-switching strategy to improve the fault-diagnosis accuracy is verified by simulation examples.

## 1. Introduction

In recent years, MASs have become a hot research topic and are widely used in transportation, multi-robot control and other fields [1,2,3]. Along with the complexity of the working environment and functionality of MAS increases, the safety and reliability of MASs have become more vulnerable, and they are prone to failure. Considering that the agents in a MAS are interacting with each other, when the agent fails, the output of the faulty agent may be deviated, then the neighbor agents of the fault agent and even the whole system will be affected. Therefore, the study of fault diagnosis of a MAS is of great importance.

In addition, the distributed structure of the MAS determines that the agent can only obtain relative information, such as the relative position and relative velocity of its neighbors, while in practice, the communication information between agents may show loss due to the influence of interference present in the real environment or the limitation of the communication range, which in turn leads to unreliability of the fault-diagnosis results of the agents. Therefore, more research is needed on how to solve the problem of decreased fault-diagnosis accuracy due to missing communication data between agents or other reasons.

In recent years, distributed fault-diagnosis methods have been the research focus of fault diagnosis for MASs [4,5,6], among which the observer-based fault-diagnosis method is one of the most commonly used fault-diagnosis methods for MASs. The authors in [7] constructed an adjustable parameter-based distributed fault estimation observer to obtain fault estimates in agents under directed graph topology. When the actuator of a MAS fails, the authors in [8] presented a novel distributed-sliding-mode fault-diagnosis scheme based on the local relative output information. The authors in [9] decomposed a MAS into three subsystems by coordinate transformation, then finite-time unknown input observers were constructed on specific agents by using the communication topology and local information, to achieve distributed fault diagnosis for MASs. What is more, using unknown input observers (UIO) to obtain relative output information between agents is also a commonly used fault-diagnosis scheme. The authors in [10] constructed a bank of finite frequency $H_{-}/H_{\infty }$ UIOs, and detected the fault in MAS by dividing the unknown input into the decoupled part and the non-decoupled part. The authors in [11] set UIO on each agent, and the distributed fault diagnosis for MAS was achieved by using the relative information between an agent and its neighbors. Authors in [12] studied the distributed cooperative fault-detection problems for a MAS, which constructed a robust $H_{\infty }$ observer on each agent, then, a residual-based distributed cooperative FD strategy was presented by using the zonotope method. The authors in [13] proposed a heterogeneous multi-agent fault-diagnosis method to realize the fault diagnosis through mutual information interaction and a given error signal threshold of a large aircraft actuation system.

Considering the complexity of MASs and the limitations of the actual environment, all the measurements may not be available to access sometimes. When the agent can only obtain relative information, such as the relative position and relative velocity, of its neighbors, the observer may not be observable [14]. In this case, observer-based fault-diagnosis methods may not be applicable. Authors in [15] presented a data-driven state prediction and fault classification method based on the BP neural network model by using the residual signal designed the threshold, and then the fault diagnosis is carried out. The knowledge-based methods analyze the system, do not require an accurate model of the system, instead using information about the system and expert knowledge in the relevant field to achieve fault diagnosis, and is applicable to many complex systems [16,17]. In this paper, a BRB-based distributed fault-diagnosis method is used in MAS. BRB is an expert system proposed by Yang et al. [18]. The BRB model can deal with obscure, incomplete or uncertain observational data, and can also achieve good results for small sample data. Therefore, by constructing a fault-diagnosis model of a MAS based on BRB, the influence of external disturbances, such as the environment on fault diagnosis, can be well avoided. At present, BRB-based fault diagnosis has achieved many research results in many fields [19,20,21]. Authors in [22] constructed the fault-diagnosis model of the hierarchical BRB, realized more efficient fault diagnosis of WSN, and used P-CMA-ES to optimize the parameters of BRB, making the results of the fault diagnosis more reliable. Authors in [23] considered both the performance of the sensor and the disturbance of the external environment, and quantified it as the property of the BRB model to realize the fault diagnosis of high-speed-train-running gear systems.

Through the analysis of existing research results, a BRB-based fault-diagnosis model for each agent is constructed by using the relative information between the agent and its neighbors. However, when the relative output information of the agent cannot be accurately obtained or the fault-diagnosis accuracy is too low, this causes the diagnosis results to no longer be reliable. Considering the case when there are multiple faulty agents in a MAS at the same time, when problems like this occur, the relative information used for fault diagnosis may be inaccurate, and the results of the fault diagnosis may be deviated. Therefore, in response to the above problems, this paper proposes a topology-switching strategy. According to the BRB-based fault-diagnosis model, the average distance between the obtained fault-diagnosis accuracy of each agent is calculated, and according to this average distance, the decrease in fault-diagnosis accuracy is divided into two cases, then the threshold is set according to the feedback. When the accuracy of the fault diagnosis meets the threshold condition, the topology structure is updated to improve the accuracy and reliability of the fault-diagnosis method.

In this work, based on BRB, a distributed fault-diagnosis model was constructed for MASs with one or multiple faulty agents. In addition, in view of the problem that the accuracy of the fault-diagnosis model decreases, a corresponding topology-switching strategy was proposed, and the main contributions of this paper are as follows:

- Firstly, a distributed fault-diagnosis model for MASs based on BRB is constructed, then by combining expert knowledge, the fault diagnosis of MAS is achieved, and the situation when multiple agents fail at the same time is considered, which makes the proposed method more general;
- Secondly, the strategy of setting the threshold based on the average distance between the accuracy of the fault diagnosis of each agent is proposed, and the feedback of the average distance of the obtained accuracy is used to analyze the fault-diagnosis accuracy of each agent and the system, then the threshold value is updated;
- Compared with the existing BRB-based fault-diagnosis methods [22,24], the problem of unreliable fault-diagnosis results due to the system being influenced by the faults or its own factors is considered, and a topology-switching strategy based on the average distance of the output diagnosis accuracy is proposed to update the topology of the agent when the threshold condition is satisfied so that the fault-diagnosis results are more reliable.

The structure of this paper can be divided into the following sections. Section 2 describes the preliminary knowledge of the MAS, and the problem to be solved in this paper is presented. Section 3 provides a description of the construction of the fault-diagnosis model. Section 4 describes the topology-switching strategy proposed in this paper. Section 5 gives a simulation experiment to validate the effectiveness and advancement of the method. Finally, some conclusions are given in Section 6.

## 2. Problem Formulation

Graph theory can intuitively represent the complex communication network of agents, with nodes representing each agent, and edges between nodes to describe the communication interaction between agents. Therefore, consider that an undirected graph expressed by G = (v, e, A) denotes the undirected graph, where v = {1,…,N} denotes the set of nodes and *N* is the number of nodes, e represents the set of edges and e ⊆ v × v ={$e_{1}$, $e_{2}$,…,$e_{N}$}. Node i,i∈v represents the agent in the MAS, and the neighbors of agent *i* are defined as $N_{i}=\left\{j\in |\left(i,j\right)\in e,i\neq j\right\}$. The communication topology of graph G is denoted by the adjacency matrix A $=\left[a_{ij}\right]\in R^{N\times N}$, and if (i,j)∈e, $\;a_{ij}=1$, else $a_{ij}=0$. The Laplacian matrix of graph G is denoted as L $=\left[l_{i,j}\right]\in R^{N\times N}$, and if i=j, then $l_{ij}=\sum_{j=1}^{N}a_{ij}$, else if $i\neq j,l_{ij}=-a_{ij}$.

When the communication topology of the MAS meets certain conditions and is thus adjusted, define the adjusted topology graph as $\bar{G}$ = ($\bar{v}$, $\bar{e}$, A), where $\bar{e}$ ⊆ $\bar{v}$ × $\bar{v}$ ={$\bar{e_{1}}$, $\bar{e_{2}}$,…,$\bar{e_{N}}$} is the set of edges and $\bar{A}$$=\left[{\bar{a}}_{ij}\right]\in R^{N\times N}$ is the adjacency matrix of the adjusted topology; then, the Laplacian matrix of $\bar{G}$ is $\bar{L}\;$$=\left[{\bar{l}}_{ij}\right]\in R^{N\times N}$.

The dynamics model with fault signals of agent *i* can be described by the following system:

(1) $$\left\{\begin{array}{c} {\dot{x}}_{i}\left(t\right)=v_{i}\left(t\right),\\ {\dot{v}}_{i}\left(t\right)=u_{i}\left(t\right)+f_{i}\left(t\right), \end{array}\right.$$

where $x_{i}\left(t\right)$ represents the position of agent $i,v_{i}\left(t\right)$ represents the velocity of agent $i,u_{i}\left(t\right)$ is the control input, and $f_{i}\left(t\right)$ is the fault signal, and when there is no fault in agent $i,f_{i}\left(t\right)=0$; otherwise, $f_{i}\left(t\right)\neq 0$. It should be noted that the number of faulty agents cannot exceed half of the total number of system agents.

Based on the relative position and velocity of agent *i*, the following consistency control protocol is proposed for agent *i*:

(2) $$u_{i}\left(t\right)=\sum_{j\in N_{i}}a_{ij}\left[h_{1}\left(x_{j}\left(t\right)-x_{i}\left(t\right)\right)+h_{2}\left(v_{j}\left(t\right)-v_{i}\left(t\right)\right)\right],$$

where $h_{1},h_{2}>0$ are controller parameters.

**Assumption** **A1.** *Both the initial graph G and the adjusted graph $\bar{G}$ are undirected and connected.*

**Definition** **1.** *For the MAS (2), if the following conditions are met,*

(3) $$\lim_{t\to \infty }∥x_{i}\left(t\right)-x_{j}\left(t\right)∥=0,\lim_{t\to \infty }∥v_{i}\left(t\right)-v_{j}\left(t\right)∥=0,i=1,2,\ldots ,N.$$

*then, we can say that this multi-agent system achieves consensus.*

Note that all notations that used in this paper are shown in Table A1 of Appendix A.

The issues to be addressed in this paper are as follows.

**Problem** **1.** *There may be multiple agents malfunctioning at the same time in MAS.*

Considering that there are multiple agents that fail at the same time, since the distributed fault diagnosis uses the relative information of the neighbor nodes for fault diagnosis, if an agent and its neighbors fail at the same time, the relative information of the neighbor nodes will be no longer reliable. At this time, the fault-diagnosis accuracy of this agent may decrease. Therefore, to ensure the accuracy and universality of the fault-diagnosis model, it is necessary to consider the case of simultaneous faults of multiple agents.

**Problem** **2.** *The accuracy of fault diagnosis is low, and the results of fault diagnosis are unreliable.*

Since MASs are limited by the actual industrial environment, and the relative information between an agent and its neighboring agents can be affected by various factors and thus have deviations, this will lead to a situation where the overall fault-diagnosis accuracy of the system is reduced or the local fault-diagnosis accuracy is reduced. The local accuracy is reduced, that is, under the premise of distributed fault diagnosis, the fault-diagnosis accuracy of individual agents is decreased, and the accuracy can be improved by adjusting the topology structure appropriately. Therefore, it is necessary to propose a topology switching strategy to improve the accuracy of fault diagnosis.

## 3. BRB-Based Distributed Fault-Diagnosis Model

**Figure 1:**
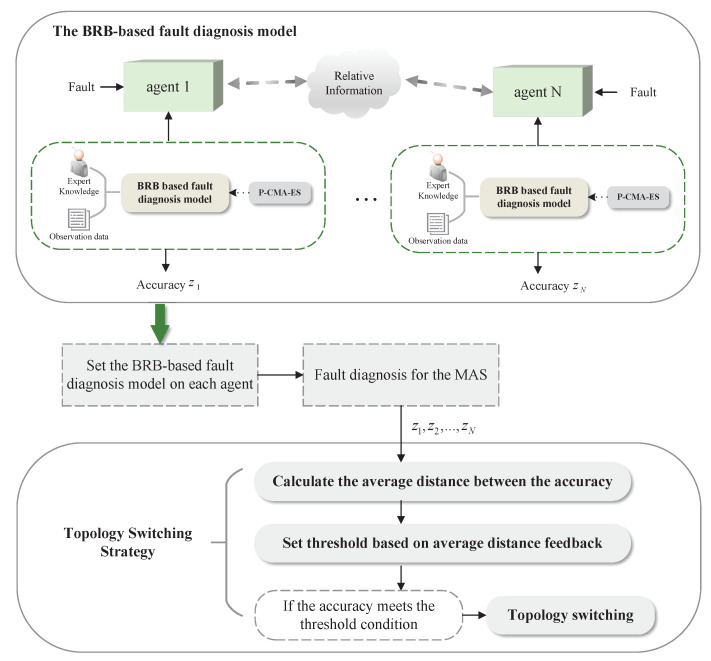
Illustration of the distributed fault-diagnosis method and the topology-switching strategy.

A BRB-based fault-diagnosis model is set up on each agent to achieve distributed fault diagnosis for MAS. Section 3.1 describes the inference calculation process of the BRB-based fault-diagnosis model on each agent; Section 3.2 describes the optimization model of the parameters in the BRB-based fault-diagnosis method. The process of the distributed fault diagnosis of MAS and the implementation of the topology-switching strategy can be clearly seen in Figure 1.

### 3.1. Inference of the BRB-Based Distributed Fault-Diagnosis Model

Considering the distributed feature of MAS, the relative information between an agent and its neighbors is used, and the consistent position error of an agent and its neighbors is calculated as the input attribute of this BRB-based fault-diagnosis model. The consistent position error of agent *i* and its neighboring agents can be obtained as

(4) $$e_{x_{i}}\left(t\right)=\sum_{j=1}^{N}a_{ij}\left[x_{i}\left(t\right)-x_{j}\left(t\right)\right],i=1,\ldots ,N,j\in N_{i}=\left\{j|\left(i,j\right)\in e\right\}.$$

The input attributes of the BRB-based fault-diagnosis model set on the agent *i* can be described as

(5) $$e_{x_{p}}^{*}=e_{x_{j}}\left(t\right),j\in N_{i},p=1,\ldots ,\left|N_{i}\right|.$$

Taking agent i,i∈[1,2,…,N] as an example, the *k* th rule of the BRB model is expressed as

(6) $$\begin{array}{c} R_{k}:\mathrm{If}\;e_{x_{1}}^{*}\;\mathrm{is}\;A_{1}^{k}\wedge \;e_{x_{2}}^{*}\;\mathrm{is}\;A_{2}^{k}\wedge \ldots \wedge \;e_{x_{\left|N_{i}\right|}}^{*}\;\mathrm{is}\;A_{\left|N_{i}\right|}^{k},\\ \;\;\mathrm{then}\;y\;\mathrm{is}\left\{\left(D_{1},\beta_{1,k}\right),\left(D_{2},\beta_{2,k}\right),\ldots ,\left(D_{Q},\beta_{Q,k}\right)\right\}\left(\sum_{q=1}^{Q}\beta_{q,k}\leq 1\right),\\ \;\;\mathrm{with}\;rule\;weight\;\theta_{k},\;attribute\;weight\;\delta_{1},\delta_{2},\ldots ,\delta_{|N_{i}|}. \end{array}$$

where $e_{x_{p}}^{*}\left(p=1,\ldots ,\left|N_{i}\right|\right)$ represents the attribute of BRB, and $A_{p}^{k}$ represents the referential value of the *p* th attribute in the *k*th rule. $\beta_{q,k}\left(q=1,2,\ldots ,Q,k=1,\ldots ,L\right)$ represents the belief degree corresponding to each consequent under the *k* th belief rule, $D_{q}$ represents the fault-diagnosis results, and *Q* is the number of statuses of agent *i* used in the *k* th rule. If $\sum_{q=1}^{Q}\beta_{q,k}=1$, the *k* th rule is considered complete. $\theta_{k}$ represents the weight of rule *k*, and $\delta_{p}\left(p=1,\cdots ,\right|N_{i}\left|\right)$ represents the attribute weight of rule *k*.

The inference steps of the BRB-based model on agent *i* are as follows.

**Step 1: Calculate the matching degree of the attributes.**

For attribute *p*, its matching degree $a_{p}^{k}$ to the reference value under the *k* th rule is calculated as follows:

(7) $$a_{p}^{m}=\left\{\begin{array}{cc} \frac{A_{p}^{\left(k+1\right)}-e_{x_{p}}^{*}}{A_{p}^{\left(k+1\right)}-A_{p}^{k}}, & \;m=k\;\mathrm{if}\;A_{p}^{k}\leq e_{x_{p}}^{*}\leq A_{p}^{\left(k+1\right)}\\ \frac{e_{x_{p}}^{*}-A_{p}^{k}}{A_{p}^{\left(k+1\right)}-A_{p}^{k}}, & \;m=k+1\\ 0, & \;m=1,2,\ldots ,\left|e_{x_{p}}^{*}\right|,m\neq k,k+1 \end{array}\right.$$

where $A_{p}^{k}$ and $A_{p}^{\left(k+1\right)}$ are the referential values of attribute *p* in the *k* th and the (k+1) th rule, respectively. This rule is activated when the matching degree of attribute *p* is not equal to 0.

**Step 2: Calculate the activation weight $w_{k}$ of the k th rule.**

In step 1, according to the matching degree, one can obtain the activated rule, and for the activated rule *k*, its activation weight $w_{k}$ can be obtained by

(8) $$w_{k}=\frac{\theta_{k}\prod_{p=1}^{M_{k}}{\left(a_{p}^{k}\right)}^{\delta_{p}}}{\sum_{k=1}^{L}\theta_{k}\prod_{p=1}^{M_{k}}{\left(a_{p}^{k}\right)}^{\delta_{p}}}.$$

**Step 3: Suppose there are k rules activated; then, use the ER algorithm to aggregate the k rules.**

(9) $$\beta_{q}=\frac{\mu \times \left[\prod_{k=1}^{L}\left(w_{k}\beta_{q,k}+1-w_{k}\sum_{q=1}^{Q}\beta_{q,k}\right)-\prod_{k=1}^{L}\left(1-w_{k}\sum_{q=1}^{Q}\beta_{q,k}\right)\right]}{1-\mu \times \left[\prod_{k=1}^{L}\left(1-w_{k}\right)\right]},$$

and

(10) $$\mu ={\left[\sum_{q=1}^{Q}\prod_{k=1}^{L}\left(w_{k}\beta_{q,k}+1-w_{k}\sum_{q=1}^{Q}\beta_{q,k}\right)-\left(Q-1\right)\prod_{k=1}^{L}\left(1-w_{k}\sum_{q=1}^{Q}\beta_{q,k}\right)\right]}^{-1},$$

where $\beta_{q}$ represents the belief degree of the *q* th consequent $D_{q}$.

**Step 4: Calculate the utility of the model.**

The output of the fault-diagnosis model can be described as

(11) $$S\left(e_{x}^{*}\right)=\left\{\left(D_{q},\beta_{q}\right),q=1,2,\ldots ,Q\right\},$$

suppose the utility of the *q* th fault-diagnosis result $D_{q}$ is $u\left(D_{q}\right)$; then, one can obtain the utility of the fault-diagnosis model by

(12) $$u\left(S\left(e_{x}^{*}\right)\right)=\sum_{q=1}^{Q}u\left(D_{q}\right)\beta_{q}.$$

### 3.2. Optimization of the BRB-Based Fault-Diagnosis Model

During the initialization of this model, the parameters defined by experts in the model are not optimal parameters of the model in many cases. The projection covariance matrix adaption evolution strategy (P-CMA-ES) is a global optimization algorithm and is suitable for dealing with a nonlinear, multi-objective optimization problem. P-CMA-ES combines the reliability and global capability of evolution strategy (ES) with the high covariance matrix of the adaptive covariance matrix, and uses the projection operation to map the candidate back to the feasible domain. Compared with CMA-ES, P-CMA-ES can solve the problem of constrained optimization, which is more suitable for the parameter optimization problem of BRB [25].

**Figure 2:**
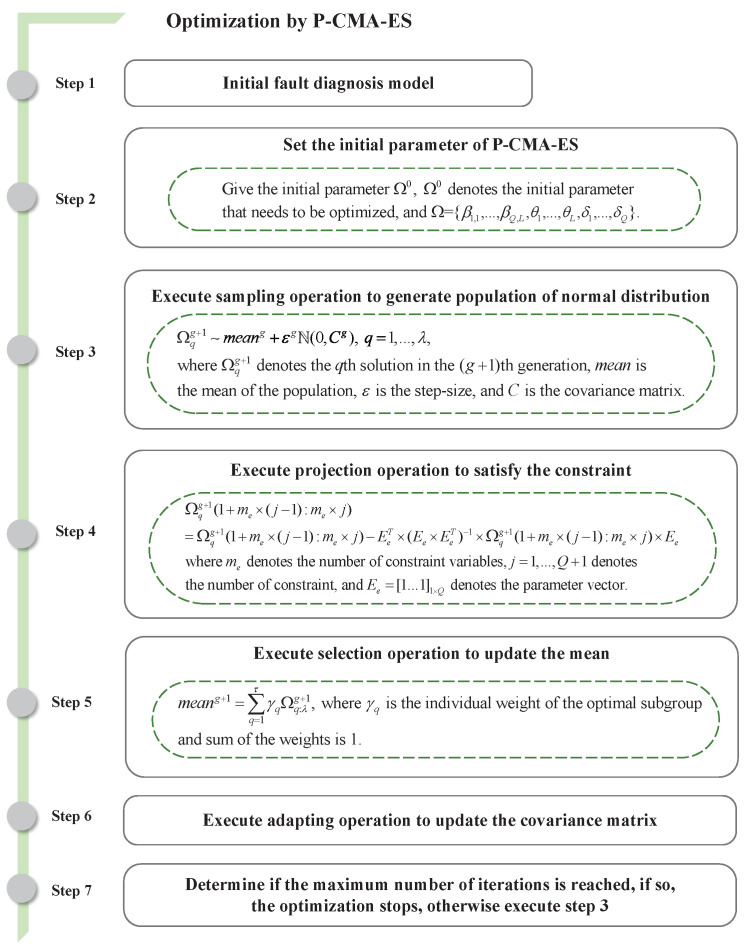
Optimization of the fault-diagnosis model.

Therefore, to solve the uncertainty of expert knowledge, this paper uses the P-CMA-ES algorithm to achieve the optimization of the parameters of this fault-diagnosis model. The objective function for the optimization of the model parameters can be defined as

(13) $$\begin{array}{cc} \; & \min MSE\left(\beta_{q,k},\theta_{k},\delta_{p}\right)\\ \; & \;s.t.\;\\ \; & 0\leq \beta_{q,k}\leq 1,k=1,\ldots ,L,q=1,\ldots ,Q\\ \; & \sum_{q=1}^{Q}\beta_{q,k}\leq 1,\\ \; & 0\leq \theta_{k}\leq 1,\\ \; & 0\leq \delta_{p}\leq 1,p=1,\ldots ,\left|N_{i}\right|, \end{array}$$

where $MSE\left(\beta_{q,k},\theta_{k},\delta_{p}\right)$ denotes the mean square error of the actual output and the expected output, which can be described by

(14) $$MSE\left(\beta_{q,k},\theta_{k},\delta_{p}\right)=\frac{1}{NUM}\sum_{\mathrm{num}\;=1}^{NUM}{\left(u_{\mathrm{actual}\;}-u_{\mathrm{expexted}\;}\right)}^{2},$$

where NUM represents the number of samples, and $u_{\mathrm{actual}\;}$ and $u_{\mathrm{expected}\;}$ represent the actual output and expected output of the fault-diagnosis model, respectively. The optimization process of the fault-diagnosis model is shown in Figure 2.

## 4. Inference of the Topology Switching Strategy

In this section, the inference of the topology switching strategy is given. In Section 4.1, the calculation process of the topology switching threshold is given; next, for MAS satisfying the threshold condition, the topology switching operation is given in Section 4.2.

### 4.1. Inference Process of the Threshold

The average distance is an indicator that reflects the central tendency of the data, which refers to the sum of all data in a set of data divided by the number of data in this set. The average distance is one of the important measures to describe the central tendency and dispersion of data samples.

For the BRB-based fault-diagnosis model set on each agent, calculate the average distance between the output fault-diagnosis accuracy, take the sum and multiply by $\frac{2}{n(n+1)}$, where *n* represents the total number of samples in the dataset, that is, the number of agents. The specific calculation process is as follows:

(15) $$\begin{array}{c} average\_distance=\frac{2}{n(n+1)}\times \sum_{i=1}^{n-1}\sum_{j=i+1}^{n}\left|z_{i}-z_{j}\right|, \end{array}$$

where $z_{1},\ldots ,z_{n}$ represents the fault-diagnosis accuracy of the BRB model set on agent *i*, and average_distance represents the average distance between the fault-diagnosis accuracy of the BRB output on each agent.

The obtained average distance of fault-diagnosis accuracy can be classified into two cases: the average distance is large and the average distance is small. Therefore, in these two cases, the factors that may lead to the decrease in fault-diagnosis accuracy are analyzed separately, and appropriate thresholds are set.

- For the case where the average distance is large, we have the following steps:

**Step 1: Analyze the possible states of the fault-diagnosis system at this time.**

When the average distance is large, it indicates that the gap between the fault-diagnosis accuracy of each agent is large, that is, the fault-diagnosis accuracy of individual agents is low, which may be caused by the fact that an agent has only one neighbor agent and the relative information is insufficient, or the neighbors of an agent are faulty, which may also lead to deviations in relative information, resulting in inaccurate fault diagnosis.

**Step 2: When the fault-diagnosis accuracy decreases, analyze the feedback of the average distance of the obtained fault-diagnosis accuracy.**

When the obtained average distance is large, it means that it is a local problem caused by the low fault-diagnosis accuracy of the individual agents. At this time, the accuracy of the agent must be sensitive to the threshold, which means that once the accuracy rate is lower than the set threshold, the topology structure should be adjusted in time to ensure the validity and reliability of fault diagnosis. Therefore, when the average distance is large, the set topology switching threshold should be appropriately larger.

**Step 3: Set a suitable threshold.**

The thresholds are set as follows:

(16) $$\begin{array}{cc} \; & \mathrm{If}\;average\_distance>\lambda \wedge \delta_{2}<z_{i}<\delta_{1},\\ \; & \mathrm{switch}\;\mathrm{the}\;\mathrm{topology}. \end{array}$$

where λ represents the judgment standard of average_distance, $\delta_{1}$ and $\delta_{2}$ respectively represent the threshold conditions to be met for topology switching, and note that $\delta_{1}>\delta_{2}$.

- For the case where the average distance is small, we have the following steps:

**Step 1: Analyze the possible states of the fault-diagnosis system at this time.**

When the average distance is small, there are two possibilities, the first is that the accuracy of the fault-diagnosis model on each agent is very high, indicating that the model is suitable for the distributed fault diagnosis of MAS and has a good effect.

The second is that the accuracy of the fault-diagnosis model on each agent is very low. At this time, the relative information between the agents may be deviated due to the overall problem of the system, and the accuracy of the fault diagnosis will be affected, or because the model is not suitable for fault diagnosing with this MAS.

**Step 2: When the fault-diagnosis accuracy decreases, analyze the feedback of the average distance of the obtained fault-diagnosis accuracy.**

When the average distance is small, the decrease in the fault-diagnosis accuracy may be a problem of the overall system. The topology-switching threshold should be low. When the accuracy rate is lower than this threshold, it means that the overall fault diagnosis of the system is no longer reliable, and other operations need to be considered.

**Step 3: Set a suitable threshold.**

The thresholds are set as follows:

(17) $$\begin{array}{cc} \; & \mathrm{If}\;average\_distance<\lambda \wedge z_{i}>\delta_{1},\\ \; & \mathrm{The}\;\mathrm{diagnosis}\;\mathrm{results}\;\mathrm{of}\;\mathrm{the}\;\mathrm{fault}-\mathrm{diagnosis}\;\mathrm{model}\;\mathrm{are}\;\mathrm{accurate};\\ \; & \mathrm{else}\;\mathrm{if}\;average\_distance<\lambda \wedge z_{i}<\delta_{2},\\ \; & \;\mathrm{perform}\;\mathrm{other}\;\mathrm{operations}, \end{array}$$

**Remark** **1.** *The threshold is set according to the fault-diagnosis accuracy of the model. A large switching threshold is intended to monitor and switch the topology in time when the accuracy of the fault-diagnosis model on an agent decreases to ensure the reliability of the fault diagnosis.*

**Remark** **2.** *After testing, for a MAS with 5 agents, when the fault-diagnosis accuracy of each agent is above 90%, the average distance will not exceed 5, and therefore, 5 can be used as a benchmark to judge whether the average distance is large or small, but the final specific value should be determined based on the experimental results.*

### 4.2. Description of the Topology Update Operation

**Figure 3:**
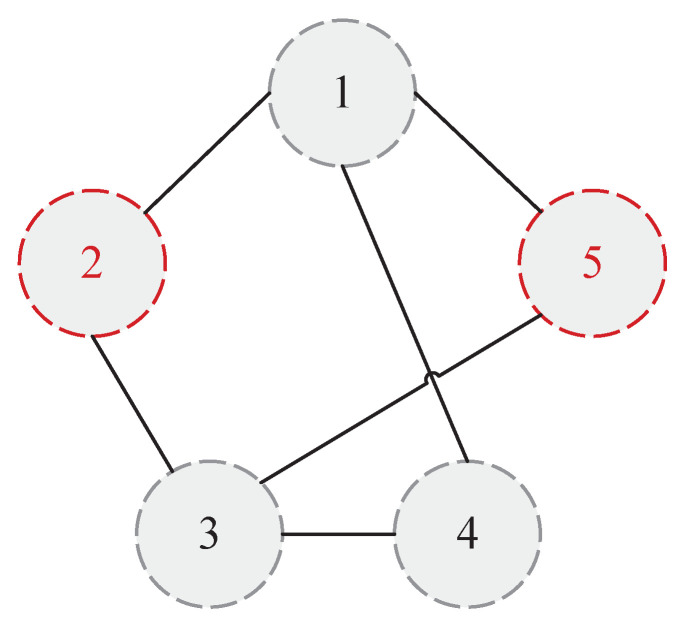
Topology of the network.

**Figure 4:**
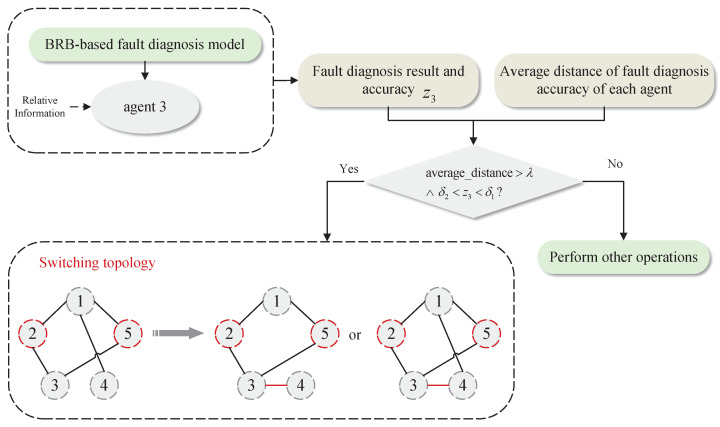
Topology-switching process for case 1.

This paper mainly analyzes the situation that the accuracy of local fault diagnosis decreases, and proposes the corresponding topology-switching strategy. Taking the MAS with 5 agents as an example, as shown in Figure 3, and assuming that agent 2 and agent 5 are faulty, for two common situations that may lead to low fault-diagnosis accuracy, the topology-switching strategy is as follows:

**Case 1:** The first is to consider the situation that there are multiple faulty agents in the MAS. At this time, if an agent and its neighbor agents fail at the same time, or all the neighbor nodes of an agent fail, it may lead to deviations in the fault-diagnosis results. It can be solved by adding or switching neighbor agents. For agent 3, it can be seen from Figure 3 that both of its neighbor agents are faulty. Therefore, for the BRB fault-diagnosis model on agent 3, the specific operations are shown in Figure 4.

**Figure 5:**
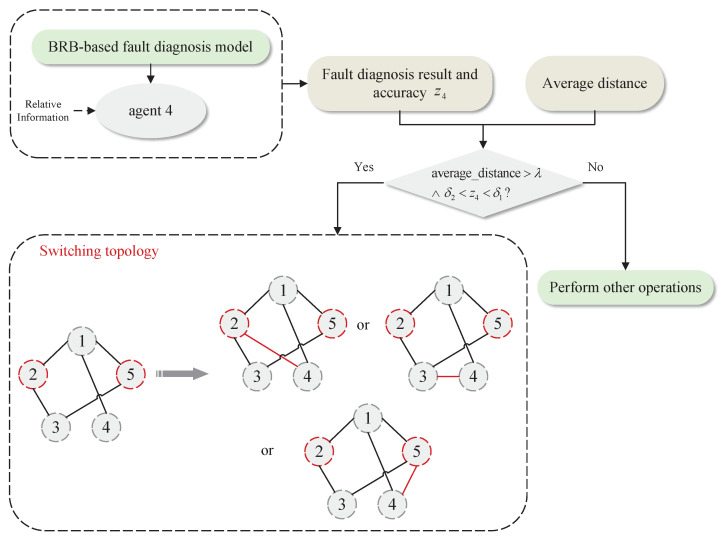
Topology-switching process for case 2.

**Case 2:** Secondly, there is a problem that an agent has insufficient relative information due to only one neighbor or other factors, which will also make the fault-diagnosis result inaccurate. At this time, the neighbor agents of the agent can be added to improve the BRB model data. The specific operation is shown in Figure 5.

**Remark** **3.** *When adding neighbor agents or adjusting the topology, the agent with a small number of neighbor agents should be selected as the new neighbor agent of an agent so as to avoid excessive computational burden on the BRB model and avoid the problem of combinatorial explosion.*

**Remark** **4.** *Compared with some existing fault-diagnosis methods, this paper gives more consideration to improving the reliability of fault-diagnosis results. This topology-switching strategy is proposed for the fault diagnosis of MAS, which can well cope with the problem of decreasing the accuracy of fault diagnosis, and improve the reliability of the fault-diagnosis methods.*

## 5. Case Study

*A. System description*

**Figure 6:**
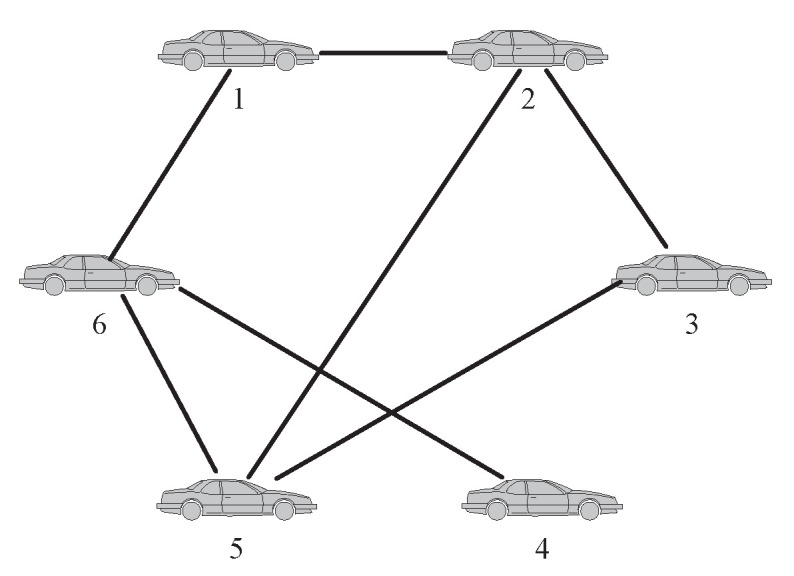
Communication topology of the multi-vehicle system.

Considering that the vehicles in a multi-vehicle system are interacting with each other, when one or multiple vehicles fail, the output of the faulty vehicle may be deviated, and the neighbor vehicles of the fault vehicles and even the whole system will be affected. Therefore, in this section, consider a multi-vehicle system with 6 vehicles shown in (18).

(18) $$\left\{\begin{array}{c} {\dot{x}}_{i}\left(t\right)=v_{i}\left(t\right),\\ {\dot{v}}_{i}\left(t\right)=u_{i}\left(t\right)+f_{i}\left(t\right), \end{array}\right.$$

where $x_{i}\left(t\right)$ and $v_{i}\left(t\right)$ represent the position and velocity of vehicle *i*, respectively, $u_{i}\left(t\right)$ is the control input, and $f_{i}\left(t\right)$ is the fault signal of vehicle *i*. The communication connection of each vehicle in the system is shown in Figure 6.

**Figure 7:**
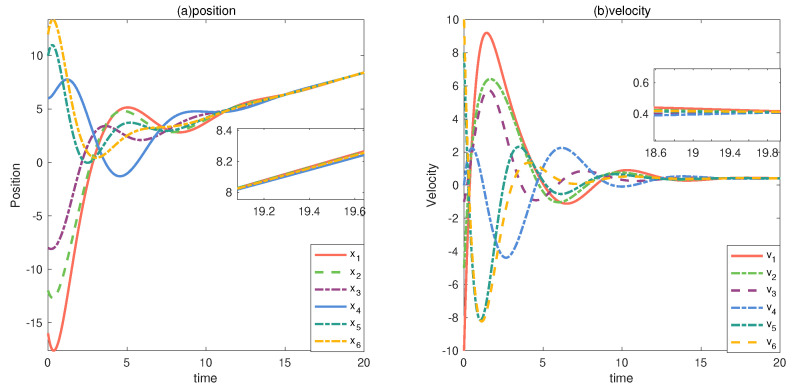
$x_{i}$ and $v_{i}$ under normal conditions.

This multi-vehicle system uses (2) as the control input, and the initial values of position and velocity of each vehicle are set as follows: $x_{1}\left(t\right)=-16,x_{2}\left(t\right)=-12,$ $x_{3}\left(t\right)=-8,x_{4}\left(t\right)=6,x_{5}\left(t\right)=10,x_{6}\left(t\right)=12,v_{1}\left(t\right)=-10,v_{2}\left(t\right)=-5,v_{3}\left(t\right)=-1,$ $v_{4}\left(t\right)=0,v_{5}\left(t\right)=8,v_{6}\left(t\right)=10$. The control parameters $h_{1}$ and $h_{2}$ in (2) are set as 0.6. As shown in Figure 7, the position and velocity of each vehicle under the control of the control law reach a consistent state over a period of time.

**Figure 8:**
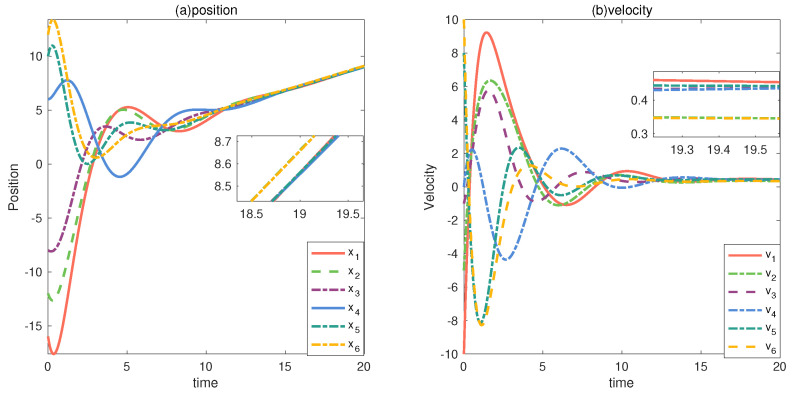
$x_{i}$ and $v_{i}$ under fault conditions.

Assume that in this multi-vehicle system, both vehicle 2 and vehicle 6 have constant faults, and the faults are described as $f_{2}\left(t\right)=0.1,f_{6}\left(t\right)=0.1$. Figure 8 shows the status of the vehicles when vehicle 2 and vehicle 6 are faulty, and from the figure, it can be clearly seen that after the system stabilizes, when vehicle 2 and 6 fail, their positions and velocities deviate from those of the vehicle that did not fail.

*B. Construction of the fault-diagnosis model*

By observing the state of each vehicle when vehicle 2 and vehicle 6 fail, select key factors as the prior attribute, according to Section 3.1, according to the relative information between the vehicle and its neighbors, and calculate the consistent position error between this vehicle and its neighbors as follows:

(19) $$e_{x_{i}}\left(t\right)=\sum_{j=1}^{N}a_{ij}\left[x_{i}\left(t\right)-x_{j}\left(t\right)\right],i=1,\cdots ,6.$$

**Figure 9:**
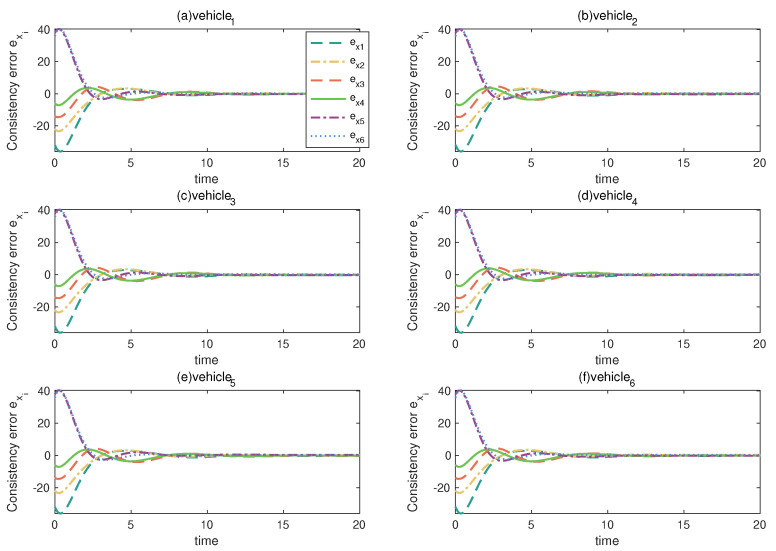
Consistent position error.

**Figure 10:**
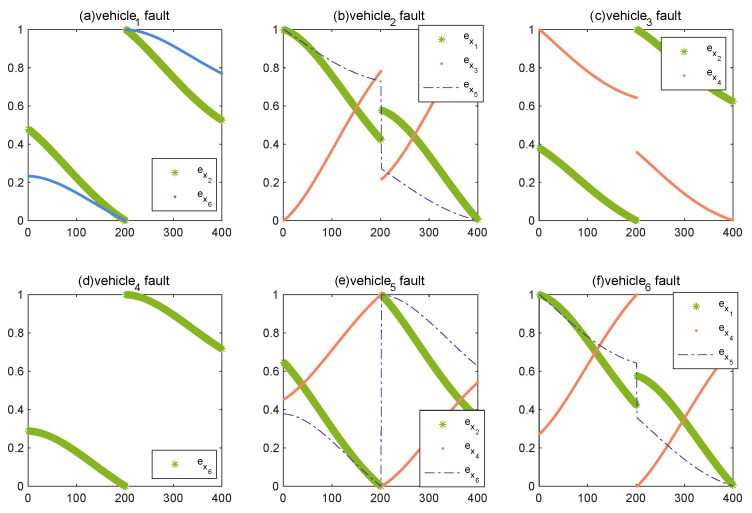
Consistent position error after normalizing.

Use the obtained consistent position error as the prior attribute of the BRB model. Figure 9 shows the consistent position error of a vehicle and its neighbors if a vehicle also fails under the premise that both vehicle 2 and vehicle 6 have a constant fault. It can be seen through Figure 9 that the difference in $e_{x_{i}}$ between the vehicle that failed and the other vehicles is not significant; therefore, in order to make the characteristics of the premise attribute more obvious, the obtained consistent position error is normalized. According to the consistent position error, after normalization as shown in Figure 10, set the reference points and reference values for the attributes and output results, and construct the belief rules of BRB, then the BRB-based fault-diagnosis model of this multi-vehicle system can be described as follows:

(20) $$\begin{array}{c} R_{k}:\mathrm{If}\;e_{1}\;\mathrm{is}\;A_{1}^{k}\wedge \;e_{2}\;\mathrm{is}\;A_{2}^{k}\wedge \ldots \wedge \;e_{6}\;\mathrm{is}\;A_{6}^{k},\\ \;\;\mathrm{then}\;y\;\mathrm{is}\left\{\left(N,\beta_{1,k}\right),\left(F,\beta_{2,k}\right)\right\}\left(\sum_{q=1}^{Q}\beta_{q,k}\leq 1\right),\\ \;\;\mathrm{with}\;rule\;weight\;\theta_{k},\;attribute\;weight\;\delta_{k_{1}},\ldots ,\delta_{k_{6}}, \end{array}$$

where $e_{i},i=1,\cdots 6$ represents the consistent position error after normalization.

According to Figure 10, take the fault-diagnosis model on vehicle 2 as an example. By combining data, the semantic values for $e_{1}$ are set as $\left[a_{1},b_{1},c_{1},d_{1}\right]$, the semantic values for $e_{3}$ are set as $\left[a_{2},b_{2},c_{2},d_{2}\right]$, the semantic values for $e_{5}$ are set as $\left[a_{3},b_{3},c_{3}\right]$, and Table 1, Table 2 and Table 3 show the reference values for each attribute. For the results of fault diagnosis, the semantic values and reference values are set as shown in Table 4, and Table 5 shows the belief rules that optimized by P-CMA-ES.

**Figure 11:**
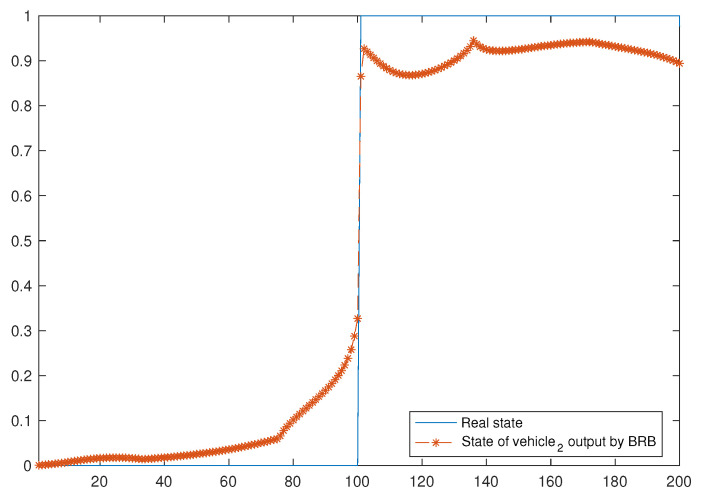
Fault-diagnosis results of $vehicle_{2}$.

When training the model, the data within [13–15] s are selected for training, and the step size is set to 0.01, so there are 200 sets of data in total. For these 200 sets of data, 100 sets are derived from the data of the vehicle and its neighbors when the vehicle is normal, and the other 100 sets are from the data of the vehicle and its neighbors when the vehicle is faulty. In addition, the number of iterations of the optimized model is set to 50. Figure 11 shows the fault-diagnosis results of the BRB model set on vehicle 2. Similarly, one can obtain the results of the fault-diagnosis model set on other vehicles; Table 6 shows the accuracy of the fault-diagnosis model set on each vehicle.

*C. Topology switching strategy*

According to Table 6, calculate the average distance of the accuracy of each vehicle’s fault-diagnosis model by the method described in Section 4.1. When n=6, the average distance is calculated as follows:

(21) $$\begin{array}{cc} average\_distance= & \frac{2}{n(n+1)}\times \sum_{i=1}^{n-1}\sum_{j=i+1}^{n}\left|z_{i}-z_{j}\right|\\ \; & =\frac{1}{21}\times \sum_{i=1}^{5}\sum_{j=i+1}^{6}\left|z_{i}-z_{j}\right|\\ \; & =10.66. \end{array}$$

Then, set $\lambda =5,\delta_{1}=70\%,\delta_{2}=90\%$; therefore, the thresholds can be set as follows:

(22) $$\begin{array}{cc} \; & \mathrm{If}\;average\_distance>5\wedge 70\%<z_{i}<90\%,\\ \; & \mathrm{Then},\;\mathrm{switch}\;\mathrm{the}\;\mathrm{topology};\\ \; & \mathrm{else}\;\mathrm{if}\;average\_distance<5\wedge z_{i}<70\%,\\ \; & \;\mathrm{perform}\;\mathrm{other}\;\mathrm{operations}, \end{array}$$

**Figure 12:**
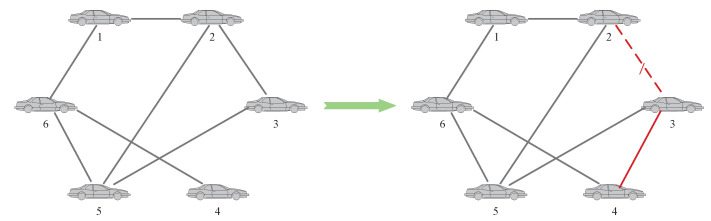
Topology switch.

It can be seen from the fault-diagnosis results of each vehicle that when vehicle 2 and vehicle 6 fail at the same time, the model diagnosis accuracy of these two vehicles is low, and the set threshold conditions are met, so the topology structure needs to be adjusted. For vehicle 2, the topology adjustment strategy is shown in Figure 12, and after topology adjustment, the fault-diagnosis accuracy of vehicle 2 reaches 99%.

**Figure 13:**
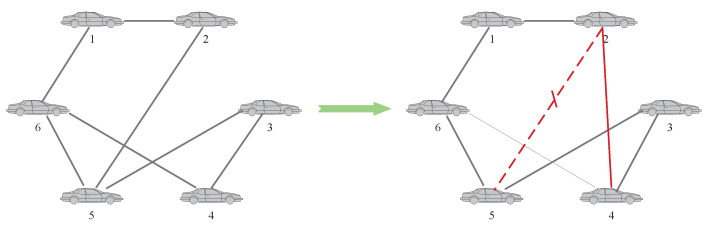
Topology switch.

Similarly, for vehicle 5, it can be seen that when vehicle 2 and vehicle 6 fail at the same time, since the neighbor nodes of vehicle 5 are vehicle 2, vehicle 6 and vehicle 3, it may be interfered by the faults of vehicle 2 and vehicle 6, resulting in a decrease in the fault-diagnosis accuracy of the BRB model on vehicle 5, which also satisfies the threshold condition and performs topology adjustment as shown in Figure 13. The adjusted fault-diagnosis accuracy rate also reaches 99%.

**Figure 14:**
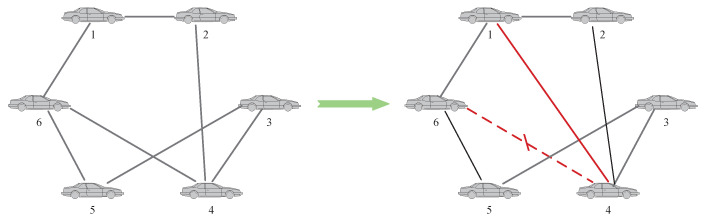
Topology switch.

Finally, for vehicle 6, topology adjustment is performed as shown in Figure 14. The adjusted fault-diagnosis accuracy rate also finally reached 99%.

**Figure 15:**
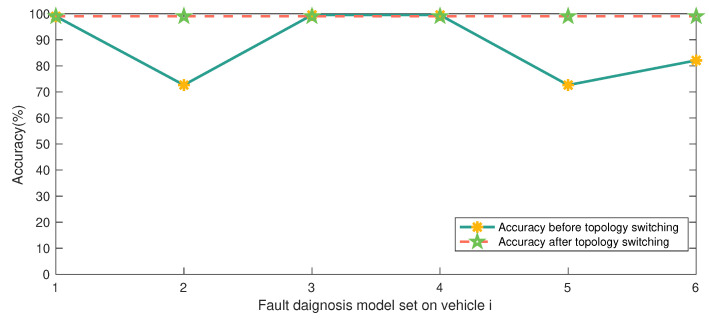
Accuracy comparison before and after topology switching.

Based on the BRB-based fault-diagnosis model for MAS, this paper proposes a topology-switching strategy by considering several common situations that may lead to unreliable fault-diagnosis results. Compared with general fault-diagnosis methods, the reliability of the diagnosis results is improved. It can be seen from Figure 15 that the accuracy of the fault-diagnosis results before and after adopting the topology-switching strategy is compared.

## 6. Conclusions

In this paper, a new topology-switching strategy for a BRB-based fault-diagnosis model of MASs is proposed to address the problem of the decreasing accuracy of the fault-diagnosis model. On the one hand, considering the limitations of model-based methods, such as observer-based methods in the actual environment, the distributed fault-diagnosis model based on BRB is constructed, and the model is made more general by taking into account the real environment where multiple fault agents may exist simultaneously in MAS. On the other hand, by analyzing the conditions that may lead to a decrease in the accuracy of the fault-diagnosis model, a strategy of using the feedback of the average distance of fault-diagnosis accuracy to set the threshold is proposed, and according to this set threshold, a topology-switching strategy is proposed to solve the problem of the decreasing model accuracy. The method proposed in this paper can well improve the reliability of the fault-diagnosis model, and the effectiveness of the proposed strategy is verified through simulation experiments applied to the multi-vehicle system.

It is worth noting that the topology-switching strategy proposed in this paper has the defect of poor adaptivity, so the subsequent research will continue to develop the topology adaptive strategy for this problem in order to further improve the fault-detection performance of the fault-diagnosis model.

## Figures and Tables

**Table 1:** Reference values for $e_{1}$.

$a_{1}$	$b_{1}$	$c_{1}$	$d_{1}$
−0.01	0.43	0.58	1.01

**Table 2:** Reference values for $e_{3}$.

$a_{2}$	$b_{2}$	$c_{2}$	$d_{2}$
−0.01	0.22	0.78	1.01

**Table 3:** Reference values for $e_{5}$.

$a_{3}$	$b_{3}$	$c_{3}$
−0.01	0.3	1.01

**Table 4:** Reference values for the fault-diagnosis results.

Normal (N)	Fault (F)
0	1

**Table 5:** Optimized belief rules.

Rule Number	$e_{1}$ and $e_{2}$ and $e_{3}$	Consequent
1	$a_{1}$ and $a_{2}$ and $a_{3}$	{0.4302, 0.5698}
2	$a_{1}$ and $a_{2}$ and $b_{3}$	{0.5381, 0.4619}
3	$a_{1}$ and $a_{2}$ and $c_{3}$	{0.2076, 0.7924}
4	$a_{1}$ and $b_{2}$ and $a_{3}$	{0.2425, 0.7575}
5	$a_{1}$ and $b_{2}$ and $b_{3}$	{0.1786, 0.8214}
6	$a_{1}$ and $b_{2}$ and $c_{3}$	{0.1516, 0.8484 }
7	$a_{1}$ and $c_{2}$ and $a_{3}$	{0.0881, 0.9119}
8	$a_{1}$ and $c_{2}$ and $b_{3}$	{0.4977, 0.5023}
9	$a_{1}$ and $c_{2}$ and $c_{3}$	{0.8801, 0.1199 }
10	$a_{1}$ and $d_{2}$ and $a_{3}$	{0.0969, 0.9031}
11	$a_{1}$ and $d_{2}$ and $b_{3}$	{0.5275, 0.4725}
12	$a_{1}$ and $d_{2}$ and $c_{3}$	{0.3316, 0.6684}
13	$b_{1}$ and $a_{2}$ and $a_{3}$	{0.5138,0.4862}
14	$b_{1}$ and $a_{2}$ and $b_{3}$	{0.3100, 0.6900}
15	$b_{1}$ and $a_{2}$ and $c_{3}$	{0.3706, 0.6294}
16	$b_{1}$ and $b_{2}$ and $a_{3}$	{0.0285, 0.9715}
17	$b_{1}$ and $b_{2}$ and $b_{3}$	{0.0818, 0.9182}
18	$b_{1}$ and $b_{2}$ and $c_{3}$	{0.4257, 0.5743}
19	$b_{1}$ and $c_{2}$ and $a_{3}$	{0.0437, 0.9563}
20	$b_{1}$ and $c_{2}$ and $b_{3}$	{0.3203, 0.6797}
21	$b_{1}$ and $c_{2}$ and $c_{3}$	{0.9063, 0.0937}
22	$b_{1}$ and $d_{2}$ and $a_{3}$	{0.6356, 0.3644}
23	$b_{1}$ and $d_{2}$ and $b_{3}$	{0.3184, 0.6816}
24	$b_{1}$ and $d_{2}$ and $c_{3}$	{0.5022, 0.4978}
25	$c_{1}$ and $a_{2}$ and $a_{3}$	{0.7498, 0.2502}
26	$c_{1}$ and $a_{2}$ and $b_{3}$	{0.5671, 0.4329}
27	$c_{1}$ and $a_{2}$ and $c_{3}$	{0.9219, 0.0781}
28	$c_{1}$ and $b_{2}$ and $a_{3}$	{0.1281, 0.8719}
29	$c_{1}$ and $b_{2}$ and $b_{3}$	{0.0000, 1.0000}
30	$c_{1}$ and $b_{2}$ and $c_{3}$	{1.0000, 0.0000}
31	$c_{1}$ and $c_{2}$ and $a_{3}$	{0.7615, 0.2385}
32	$c_{1}$ and $c_{2}$ and $b_{3}$	{0.5432, 0.4568}
33	$c_{1}$ and $c_{2}$ and $c_{3}$	{1.0000, 0.0000}
34	$c_{1}$ and $d_{2}$ and $a_{3}$	{ 0.6242, 0.3758}
35	$c_{1}$ and $d_{2}$ and $b_{3}$	{0.7418, 0.2582}
36	$c_{1}$ and $d_{2}$ and $c_{3}$	{0.6225, 0.3775}
37	$d_{1}$ and $a_{2}$ and $a_{3}$	{0.5744, 0.4256}
38	$d_{1}$ and $a_{2}$ and $b_{3}$	{0.5234, 0.4766}
39	$d_{1}$ and $a_{2}$ and $c_{3}$	{0.8707, 0.1293}
40	$d_{1}$ and $b_{2}$ and $a_{3}$	{0.6131, 0.3869 }
41	$d_{1}$ and $b_{2}$ and $b_{3}$	{0.8140, 0.1860}
42	$d_{1}$ and $b_{2}$ and $c_{3}$	{0.8507, 0.1493}
43	$d_{1}$ and $c_{2}$ and $a_{3}$	{0.4798, 0.5202}
44	$d_{1}$ and $c_{2}$ and $b_{3}$	{0.5769, 0.4231}
45	$d_{1}$ and $c_{2}$ and $c_{3}$	{0.6899, 0.3101}
46	$d_{1}$ and $d_{2}$ and $a_{3}$	{0.7602, 0.2398}
47	$d_{1}$ and $d_{2}$ and $b_{3}$	{0.4859, 0.5141}
48	$d_{1}$ and $d_{2}$ and $c_{3}$	{0.3661, 0.6339}

**Table 6:** Accuracy of fault-diagnosis model on each vehicle.

$z_{1}$	$z_{2}$	$z_{3}$	$z_{4}$	$z_{5}$	$z_{6}$
99.005 %	72.639%	99.502%	99.502%	72.617%	82.090%

## Data Availability

Not applicable.

## Abbreviations

The following abbreviations are used in this manuscript:

MAS	Multi-agent system
BRB	Belief rule base
P-CMA-ES	Projection covariance matrix adaption evolution strategy
MSE	Mean square error

## Appendix A

Table A1 shows the description of mathematical notations that used in this paper.

**Table A1:** Description of mathematical notations.

Symbols	Description
R	real number system
$R^{n}$	n-dimensional Euclidean space
$R^{m\times n}$	set of m × n-dimensional real matrices
G, $\bar{G}$	undirected graph
A, $\bar{A}$	adjacency matrix
L, $\bar{L}$	Laplacian matrix
∈	element of, belongs to
⊆	superset
|a|	the absolute value of the scalar a
|A|	the number of elements of set A
A×B	set of all ordered pairs from A and B
∥·∥	norm of a vector or matrix
∑	summation
min	minimum
